# Segmental Isotope Labelling of an Individual Bromodomain of a Tandem Domain BRD4 Using Sortase A

**DOI:** 10.1371/journal.pone.0154607

**Published:** 2016-04-29

**Authors:** Felix P. Williams, Alexander G. Milbradt, Kevin J. Embrey, Romel Bobby

**Affiliations:** Discovery Sciences, Innovative Medicines and Early Development Biotech Unit, AstraZeneca, Alderley Park, Macclesfield SK10 4TF, United Kingdom; George Washington University, UNITED STATES

## Abstract

Bromodomain and extra-terminal (BET) family of proteins are one of the major readers of epigenetic marks and an important target class in oncology and other disease areas. The importance of the BET family of proteins is manifested by the explosion in the number of inhibitors against these targets that have successfully entered clinical trials. One important BET family member is bromodomain containing protein 4 (BRD4). Structural and biophysical studies of BRD4 are complicated by its tertiary-structure consisting of two bromodomains connected by a flexible inter-domain linker of approximately 180 amino acids. A detailed understanding of the interplay of these bromodomains will be key to rational drug design in BRD4, yet there are no reported three-dimensional structures of the multi-domain BRD4 and NMR studies of the tandem domain are hampered by the size of the protein. Here, we present a method for rapid Sortase A-mediated segmental labelling of the individual bromodomains of BRD4 that provides a powerful strategy that will enable NMR studies of ligand-bromodomain interactions with atomic detail. In our labelling strategy, we have used U-[^2^H,^15^N]-isotope labelling on the C-terminal bromodomain with selective introduction of ^13^CH_3_ methyl groups on Ile (δ1), Val and Leu, whereas the N-terminal bromodomain remained unlabelled. This labelling scheme resulted in significantly simplified NMR spectra and will allow for high-resolution interaction, structure and dynamics studies in the presence of ligands.

## Introduction

Bromodomain containing protein 4 (BRD4) is a reader of epigenetic marks and a key target in oncology [1, 2], inflammation [3, 4] and cardiovascular disease [5] with several inhibitors currently in clinical trials [6–9]. Bromodomains are small binding modules that interact with N –acetyl-lysines on histone tails of chromatin [10–12] and regulate gene transcription as a component of the positive transcription elongation factor b (p-TEFb) [13]. The two bromodomains in BRD4, i.e. BD1 (herein referred to as BRD4(1)) and BD2 (herein referred to as BRD4(2)), are connected by a long inter-domain linker of approximately 180 residues.

As interaction with both histones and p-TEFb is mediated by the tandem bromodomains in BRD4 [13], a proper understanding of the tandem domain, BRD4(1, 2), is key to fully understanding the mechanistic details of transcriptional regulation which forms the basis for rational drug design. Structural investigations with isolated domains will be deprived of domain-domain interactions that may be present in a multi-domain protein. It has recently been reported that in the absence of phosphorylation at BRD4’s N-terminal cluster of phosphorylation sites (NPS), the bromodomains of BRD4 remain in a binding inhibited state [14], indicating that domain-domain interactions are critical for cellular function.

Nuclear magnetic resonance (NMR) spectroscopy is able to provide unique insight into the structure, dynamics and molecular interactions of biological macromolecules. Unfortunately, liquid-state NMR spectroscopy is usually applied only to small to medium sized biological macromolecules, as both the sensitivity and ease of analysis of NMR experiments is decreased with increasing size of the biological macromolecule.

At present, NMR studies have been carried out using isolated BRD4(1) or BRD4(2) domains [15–17] but not in a multi-domain construct, presumably due to the high chemical shift degeneracy in the ^1^H-^15^N NMR spectra caused by the long and flexible inter-domain linker which complicates resonance assignments and interpretation. Therefore, protein structure, dynamics and ligand interaction studies would be complicated. Hence, incorporation of an unlabelled majority of amino acids into an isotopically labelled target protein offers a potential solution to this difficulty. While sensitivity in NMR experiments can be improved by partial or uniform deuteration of the protein [18] and the design of optimised pulse programs [19], spectral crowding has been addressed by the means of reducing the number of NMR active nuclei to simplify the spectra [20]. The latter can be achieved by moving away from uniform isotope labelling of the protein of interest. For instance, by selectively incorporating isotope-labelled amino acids, which will be active during NMR experiments with unlabelled amino acids being left undetected. These selective labelling techniques, which are restricted to labelling of only a few amino acid types in any one sample, simplify NMR spectra but are impractical for BRD4 where interactions at two highly homologous single binding domains are to be observed. A more practical approach to remove unwanted signals is to use segmental isotope labelling methods [20], which have been used successfully in the past to isotopically label single domains in multi-domain proteins and these methods have been shown to be useful in interpretation of structural and dynamics information [21, 22]. In comparison to selective amino acid labelling techniques, segmental labelling is an efficient method to remove the undesirable peaks from the flexible inter-domain linker and yet providing high-quality spectra of the structured domains.

**Fig 1:**
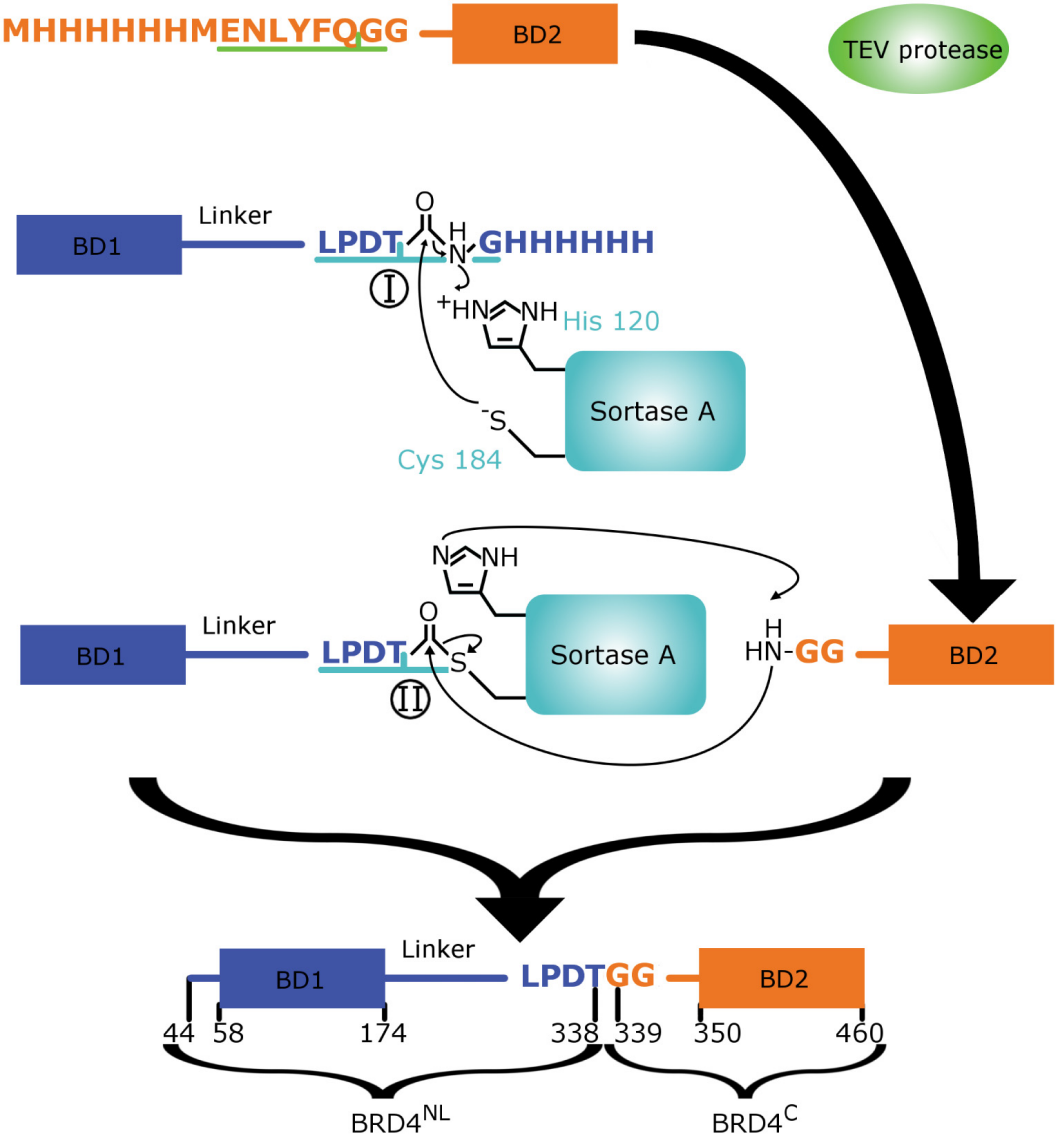
Schematic illustration of segmental labelling on BRD4(1, 2) using SrtA. Isotopically labelled BRD4^C^ (orange) was ligated to unlabelled BRD4^NL^ (blue) to give a segmentally labelled BRD4(1, 2) fusion. TEV protease cleavage and Sortase A ligation reactions were carried out separately. The two step reaction mechanisms of SrtA is also illustrated here. Step I involves an initial nucleophilic attack of the C-terminal LPDTG recognition sequence by Cys184 of SrtA. In step II there is a subsequent nucleophilic attack by the N-terminal Gly of the BRD4^C^ construct.

In this study, two constructs were expressed in a manner that allowed segmental isotope labelling of the C-terminal bromodomain in BRD4(1, 2) through a *Staphylococcus aureus* transpeptidase Sortase A (SrtA)-mediated ligation. SrtA has been comparatively little used as a segmental isotope labelling tool [20, 23] but proved to be a fast and flexible method for BRD4 protein ligation. SrtA-mediated ligation requires a recognition sequence motif LPXTG towards the C-terminus of substrate proteins. The SrtA active site cysteine cleaves the substrate protein between the Thr and Gly residues of the sortase-recognition motif and subsequently an amide bond is formed joining the newly-formed C-terminal Thr carboxylate to an amino group of a polypeptide with multiple glycines at the N-terminus (Fig 1).

As part of this study, the C-terminal bromodomain BRD4(2) was uniformly [^2^H,^15^N]-labelled with selective ^13^C-methyl protonation on Isoleucine 1, Leucine and Valine residues in a BRD4(1, 2) construct (in this construct the labelled C-terminal fragment is referred to as BRD4^C^ while the unlabelled N-terminal bromodomain and linker fragment is referred to as BRD4^NL^). This construct was successfully used to study binding of a small-molecule inhibitor to BRD4(2).

## Materials and Methods

### Constructs

The constructs used for segmental labelling of human BRD4(1, 2) were: an N-terminal construct comprising the BRD4 sequence ranging from N44 to D334 followed by the *S. aureus* transpeptidase Sortase A (SrtA) recognition sequence (LPDTG) and a hexahistidine sequence. After SrtA-mediated ligation this construct will give the N-terminus plus linker segment of the ligated BRD4 (BRD4^NL^)(Fig 1). The C-terminal construct for SrtA-mediated ligation comprised the BRD4 sequence ranging from H341 to E460 preceded by a hexahistidine tag, tobacco etch virus (TEV) recognition sequence (ENLYFQG) and a non-native glycine at codon 340. Following TEV cleavage the construct is left with a di-glycine at its N-terminus, which is required for subsequent SrtA-mediated ligation giving BRD4^C^ (Fig 1). The ligated BRD4(1, 2) construct results in mutations V335L, S338T, Q339G and Q440G.

### Protein expression and purification

All constructs were cloned into pET28 vectors (Novagen). The C-terminal domain was used to produce samples of U-[^2^H, ^13^C, ^15^N]-labelled and U-[^2^H, ^15^N]-labelled with ^13^CH_3_ labelling at the methyl groups of Isoleucines 1, Leucines and Valines (referred to as ILV labelled).

All BRD4 constructs were overexpressed in *Escherichia coli* BL21 Gold (DE3) cells. Terrific Broth (TB) was used for non-isotope enriched expression (BRD4^NL^), whereas isotope-enriched (^2^H, ^13^C, ^15^N) constructs were expressed in a D_2_O M9 medium containing 1.0 g/L ^15^NH_4_Cl and 2.0 g/L perdeuterated ^13^C glucose-d7 supplemented with 5.0 g/L [^2^H, ^13^C, ^15^N] Celtone powder (Cambridge Isotope Laboratories). For ILV labelling, the D_2_O M9 media were supplemented with 70 mg/L 2-ketobutyric acid-4-^13^C sodium salt hydrate (Isotec) and 120 mg/L 2-keto-3-(methyl-^13^C)-butyric-4-^13^C acid sodium salt (Isotec) 30 min prior to induction [24]. All expression media contained 50 μg/mL kanamycin for bacterial selection and recombinant expression was induced with 1 mM isopropyl –D-1-thiogalactopyranoside (IPTG) at an OD_600_ of 0.6 and growth continued overnight at 18°C.

Cells were pelleted, frozen at −80°C, and resuspended in 50 mM Tris pH 8, 300 mM NaCl, 10 mM β-mercaptoethanol, cOmplete protease inhibitor tablets (Roche) and 2500 U/L Benzonase nuclease. Resuspended cells were lysed using a Constant Systems cell disruptor at 25 kpsi and clarified by centrifugation at 34,000 × g for 30 min at 4°C. His-tagged BRD4 constructs were purified from supernatant by affinity chromatography using Ni-NTA beads (Sigma-Aldrich). The BRD4^NL^-Gly-His_6_ construct was further purified by cation exchange chromatography before dialysis into sortase reaction buffer (see below) for storage.

Isotope-enriched his-tagged BRD4^C^ constructs were prepared for sortase ligation by TEV cleavage of their His-tag carried out overnight at 4°C in 50 mM Tris pH 7.5, 150 mM NaCl, and 1 mM tris(2-carboxyethyl)phosphine (TCEP).

### Sortase constructs, expression and purification

*S. aureus* transpeptidase Sortase A (SrtA) used in this study comprised the sequence ranging from Q60 to K206 with single-point mutations at codons P94R, E105K, E108Q, D160N, D165A, K190E and K196T followed by a hexahistidine tag C-terminal of K206. The SrtA heptamutant exhibits higher activity [25] and is active in the absence of Ca^2+^[26]. The gene was cloned into a pET28 plasmid (Novagen).

SrtA was recombinantly expressed using the same conditions as BRD4^NL^. His-tagged SrtA was affinity purified using Ni-NTA beads (Sigma-Aldrich) and the purest fractions were pooled together as confirmed by SDS-PAGE analysis. A final dialysis into 50 mM Hepes pH 7.5, 150 mM NaCl, 1 mM TCEP, 10% (v/v) glycerol was performed and subsequently concentrated stocks of SrtA were kept at −80°C for long-term storage.

### Sortase reactions

Sortase reactions were carried out in 50 mM Tris pH 7.5, 150 mM sodium chloride and 1 mM TCEP at room temperature. Sortase reactions were carried out in separate 1 mL volumes then pooled. Concentrations used for the reactions were 36 μM BRD4^C^ domain, 18 μM BRD4(44–338) and 18 μM SrtA. Where appropriate, quenching was carried out by adding 20 mM EDTA and cooling to 4°C. The segmentally labelled product was purified by cation exchange chromatography carried out at 4°C.

### NMR spectroscopy

NMR spectra were recorded at BRD4 concentrations between 25 μM and 120 μM in a 20 mM sodium phosphate buffer at pH 6.7, 1 mM TCEP and 90%/10% (v/v) H_2_O/D_2_O. All measurements were carried out at 303 K on Bruker Avance I 600 MHz (14.1 T) or Avance III 800 MHz (18.8 T) spectrometers running TopSpin v2.1 or v3.2.5, respectively, and equipped with 5 mm z-gradient ^1^H/^13^C/^15^N TCI cryoprobes.

The 2D ^1^H-^15^N TROSY [27] experiments were acquired with spectral widths of 1733.70 Hz (^15^N) and 10162.60 Hz (^1^H). 90 × 2048 complex points were collected with 48 and 560 transients per increment recorded for the uniformly and segmentally labelled samples, respectively. The recycling time was set to 1.2 s.

The 2D ^1^H-^13^C SOFAST-methyl-TROSY [28, 29] experiments were acquired with spectral widths of 7547.2 Hz (^13^C) and 8389.3 Hz (^1^H) for the segmentally labelled BRD4(1, 2) (750 × 1024 complex points) with 400 transients per increment, 4528.0 Hz (^13^C) and 8389.3 Hz (^1^H) for the isolated BRD4(2) (110 × 1024 complex points) with 40 transients per increment, 7547.2 Hz (^13^C) and 8389.26 Hz (^1^H) for unlabelled BRD4(44–338) (110 × 1024 complex points) with 400 transients per increment. The recycle times were set to 0.1 s.

2D ^1^H-^13^C SOFAST-methyl-TROSY experiments acquired as part of the I-BET762 titration had spectral widths of 5230.1 Hz (^13^C) and 12019.2 Hz (^1^H) for the measurements carried out in the absence of I-BET762 (250 × 1024 complex points) with 1600 transients per increment. For spectra acquired in the presence of I-BET762 spectral widths were 4025.8 Hz (^13^C) and 12019.2 Hz (^1^H) (80 × 1024 complex points) with 4096 transients per increment. The recycle times were 0.001 s.

Proton chemical shifts were referenced according to sodium-2, 2-dimethyl-2-silapentane-5-sulphonate (DSS), whereas the ^15^N and ^13^C chemical shifts were indirectly referenced according to the ratios given by Markley et al. [30]. NMR spectra were processed using NMRPipe [31] and analysed in ccpnNmr Analysis v2.1.5 [32]. Figures containing NMR spectra were created using the Python based nmrGlue program [33].

## Results and Discussion

### Sortase A-mediated ligation of BRD4(1, 2)

*S. aureus* Sortase A-mediated ligation occurs between two polypeptides, where one contains a LPXTG recognition sequence motif within a few residues from the C-terminus [34, 35], X is usually D, E, N, A, Q or K [36], and a partner substrate with one or multiple glycines at its N-terminus. Ligation occurs according to a two-step mechanism in which SrtA cleaves the LPXTG recognition sequence between the Thr and Gly peptide bond. The cleavage product C-terminal of the Gly is then released while the remainder is covalently bound to the enzyme (Fig 1). The ligation partner can subsequently be attached through nucleophilic attack by the amide group of its N-terminal glycine (Fig 1). Increasing the number of N-terminal glycines on the C-terminal sequence improves efficiency but gains are small beyond two [37].

It is highly advantageous to put the LPXTG sequence in an unstructured region of the multi-domain protein which will facilitate access for SrtA to the recognition site. In addition, restricting the recognition sequence into a linker region will likely have minimal effect to the overall fold of the bromodomains and increase the likelihood for successful expression of the fragments in a recombinant system. Fortunately, the bromodomains in BRD4(1, 2) are separated by a long inter-domain linker of approximately 180 residues, which provides ample possibilities to insert the SrtA LPXTG motif. Therefore, the choice for the LPXTG insertion in the BRD4(1, 2) sequence relied on minimising the introduction of total number of mutations while keeping the position close to the C-terminal bromodomain. Residues V335, P336, D337, S338 and Q339 in wildtype BRD4(1, 2) were chosen to be an ideal location for the insertion of the SrtA recognition sequence, as the favourable aspartate can be placed at the X position and the native P336 remains after the insertion. Residues 335–340 are located towards the C-terminus of the inter-domain linker and in close proximity to the C-terminal bromodomain (starting at K349). In addition, the two residues directly following the insertion sequence, i.e., Q339 and Q340, were mutated to glycines in order to improve ligation efficiency [38]. After successful SrtA catalysis, the product is BRD4(1, 2) [V335L, S338T, Q339G, Q440G].

In order to segmentally isotope label BRD4(2), the tandem domain BRD4(1, 2) was expressed as two fragments. The unlabelled N-terminal fragment (BRD4^NL^) comprised BRD4 residues N44-K57, the N-terminal bromodomain (R58-L174), and the inter-domain linker residues P178-D334 followed by the SrtA LPDTG recognition sequence, whereas the labelled C-terminal fragment (BRD4^C^) comprised a di-glycine followed by 10 residues of the C-terminal inter-domain linker (H340-K349) and the C-terminal bromodomain (V350-E460) (Fig 1).

The BRD4^NL^ construct was expressed with a C-terminal hexahistidine tag, which is removed by SrtA during ligation (Fig 1). The BRD4^C^ construct was prepared by removal of its hexahistidine tag by TEV cleavage (See Materials & Methods). Interestingly, this cleavage reaction can be run simultaneously to the ligation reaction (S1 Fig), although, we did not carry out subsequent reactions in this fashion in order to maintain better control over our ligation reaction.

Following preliminary testing of different concentrations and molar ratios of precursors, a 1:2:1 ratio was selected for BRD4^NL^, BRD4^C^ and SrtA, respectively, at 18 μM concentration of BRD4^NL^. Increasing the ligation temperature has been suggested to speed up the reaction [39] but can also accelerate degradation of the precursors [40]. To minimise the losses all experiments were carried out at ambient temperature.

**Fig 2:**
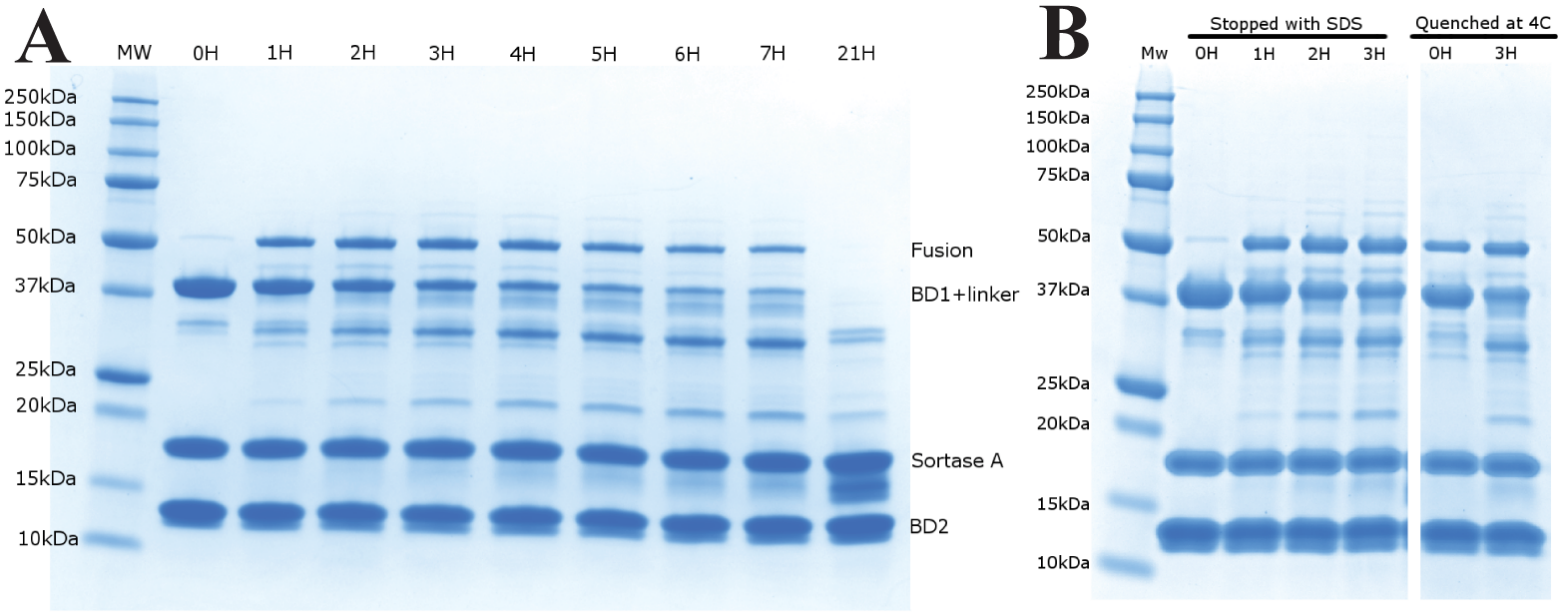
SDS-PAGE analysis of Sortase A-mediated ligation. (A) 36 μM BRD4^C^ and 18 μM BRD4^NL^ were reacted using 18 μM SrtA in 50 mM Tris (pH 7.5), 150 mM NaCl and 1 mM TCEP over the course of 21 h. Samples were taken at the timepoints shown and reaction was stopped by addition of SDS running buffer followed by denaturation at 90°C. (B) Illustration of the slow quenching effect by addition of SDS and denaturation at 90°C at timepoints 0 h, 1 h, 2 h and 3 h (left) compared to quenching at 0 h and 3 h timepoints by addition of 20 mM EDTA at 4°C (right).

Several pHs for the reaction were tested showing optimal yields between pH 7.0 and 8.0 (S1 Table), subsequent reactions were therefore carried out at pH 7.5. When carried out in a closed system our ideal reaction time was found to be roughly 1.5 h (Fig 2A), followed by quenching by addition of 20 mM EDTA and cooling to 4°C as previously described [41, 42]. The quenching of the reaction takes place slowly over the course of up to an hour (Fig 2B) which is partly due to slow cooling of the reaction. In order to keep this cooling time constant all large scale reactions were carried out in multiple small reaction volumes run in parallel and quenched for at least 30 minutes at 4°C before ion exchange purification which was also carried out at 4°C.

Our reaction was particularly fast when compared to rates reported in the literature [39–41, 43, 44]. This might be due to the inter-domain linker being particularly long and unstructured. SrtA peptide-peptide ligation is faster than protein-protein ligation and this is hypothesised to be due to diminished steric hindrance [37]. Therefore binding to a more extended and flexible linker region as in BRD4 might have similar effects by mimicking a peptide-peptide ligation.

Recently, a new method for SrtA-mediated ligation was proposed by Freiburger et al. [40], wherein the enzymatic ligation reactions were carried out in an open system with continuous removal of the cleaved peptide fragments through centrifugal filtration. In order to improve ligation efficiencies this method was assessed by carrying out SrtA reactions in 10 kDa molecular weight cut-off concentrators and centrifuging at 2000 × g for cycles of 10 min with buffer additions in between [40]. By this method, the cleaved Gly-hexahistidine peptide (Fig 1) was constantly removed from the reaction mixture.

As expected, the centrifuge-based open system drove the ligation reaction. This was assumed to be due to a mass action effect as previously suggested [41, 43], but may also be the result of transiently higher concentrations of substrates and enzyme in the concentrator.

Despite the achieved reaction times, the closed as well as the open system showed slow degradation of our fusion product over time (Fig 2A). Given that this degradation is missing in the absence of SrtA and ceases when the fusion product is separated from SrtA by chromatography, it is plausible that the degradation is caused by SrtA-mediated hydrolysis of the fusion product’s SrtA recognition site (LPDTG). This would be due to attack by water at the carboxy group of the threonine in the absence of any amino group nucleophile partner as reported in Ton-That et al. [45].

The degradation effect also complicated purification of the fusion product as the N-terminal by-product of this reaction has a molecular weight and a theoretical isoelectric point that is similar to that of the fusion product (33 kDa vs 47 kDa and 9.55 vs 9.10, respectively). As the unlabelled BRD4^NL^ fragment makes up 71% of the sequence of the final fusion product, it is anticipated that its physiochemical properties have a strong influence on those of the final product. Unlike unreacted BRD4^NL^ this by-product cannot be separated by nickel affinity chromatography as the hexahistidine tag is cleaved off during earlier reactions. The two proteins could not be separated by size exclusion chromatography and although we used ion exchange chromatography, separation was poor leading to low yields during the final purification step. In future work on this system we would apply a tagging strategy similar to that described in Freiburger et al. [40] in which the final product would contain a cleavable tag on the C-terminus in order to aid separation.

The improvements in the open system notwithstanding, this method also intensified the degradation of the reaction product BRD4(1, 2), presumably through a similar effect as that observed in the ligation reaction (S2 Fig). Consequently, this resulted to a narrower window during which the yield was optimal but concurrently improved the yield over that of the closed system (S2 Fig).

As a means to slow down the degradation of the reaction product, a change of solvent from H_2_O to D_2_O was explored. Nucleophilic attack by D_2_O occurs more slowly than H_2_O [46], which could possibly slow the hydrolysis of the fusion protein in the absence of an amino nucleophile. Interestingly, small improvements to the yields in D_2_O compared to H_2_O seem to have occurred when the reaction was done on the bench, however, these improvements were not apparent when the centrifuge-based open system was used (S3 and S4 Figs), though degradation of the reaction product, i.e. BRD4(1, 2), was slowed under these conditions. These results are consistent with the catalysis model proposed by Ton-That et al. [45], with the kinetic isotope effect of D_2_O slowing the degradation of the fusion product. Although more subtle effects are possible such as slower enzyme dynamics due to the higher viscosity of D_2_O compared to H_2_O.

These optimisations led to yields of ligated BRD4 of roughly 40% for the reaction as quantified by comparing the SDS-PAGE Coomassie stained band intensity of the reaction limiting product before the start of the reaction to that of the band intensity of the product once the reaction was stopped. Calculations factored in the assumption that Coomassie staining intensity per mole of protein is proportional to the number of positive charges present on the protein [47] (a similar result is obtained if staining is assumed to be proportional to molecular weight). Although the yields for the SrtA-mediated ligation is lower than what has been reported in the past [39, 40, 43], as many reactions can be done in parallel, both the open system and closed system methods give sufficient amounts of segmentally labelled BRD4(1, 2) for high-resolution NMR studies.

### Segmental isotope labelling to alleviate resonance overlap and enable full residue-specific analysis of bromodomains in BRD4

**Fig 3:**
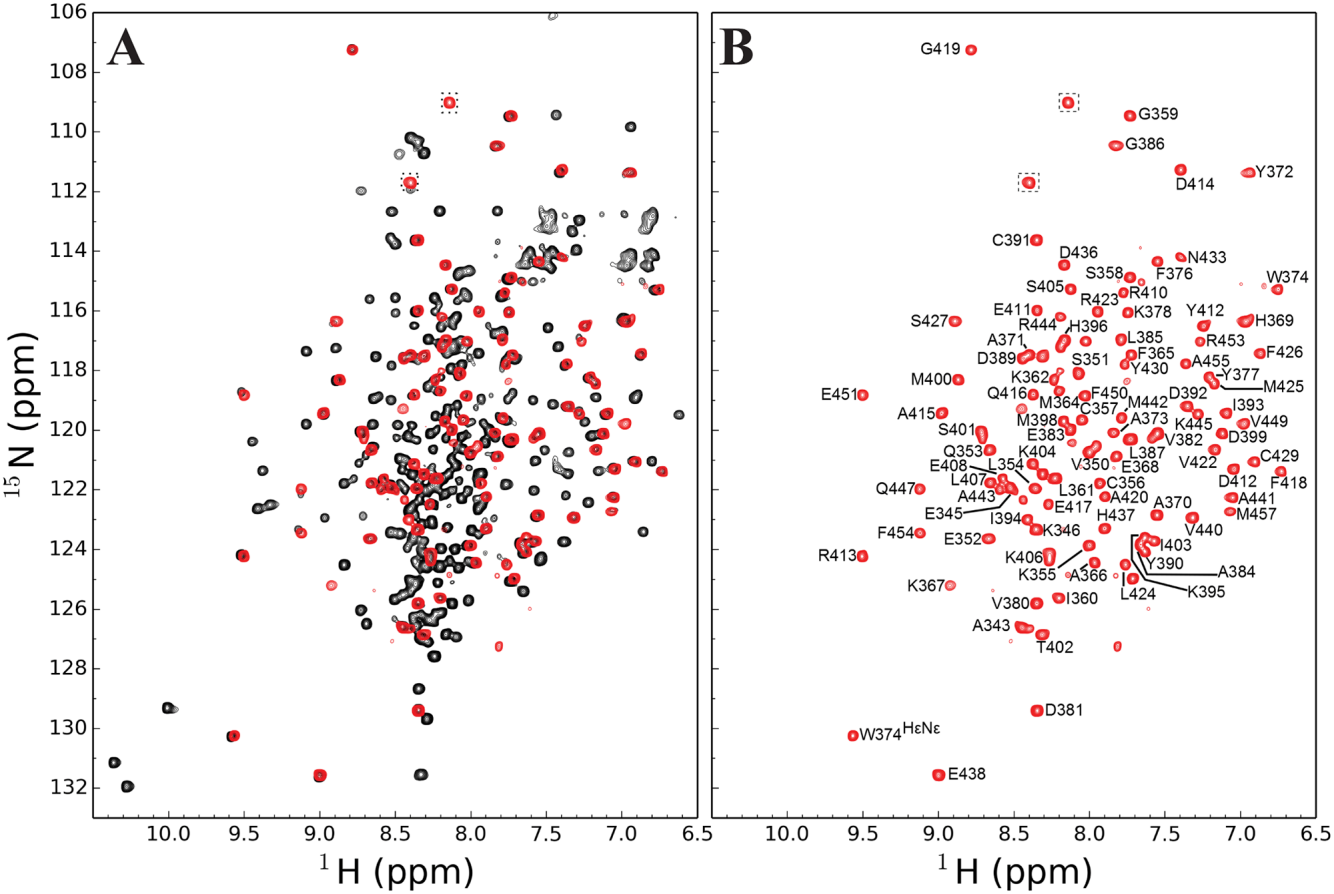
Overlay of ^1^H-^15^N TROSY HSQC spectra of uniformly and segmentally labelled BRD4. (A) ^1^H-^15^N TROSY HSQC of U-[^2^H,^15^N]-labelled BRD4(1, 2) (black) overlaid with the segmentally labelled BRD4(1, 2) with U-[^2^H,^15^N] and ^13^CH_3_ ILV methyl labelled BRD4^C^ (red). The peaks corresponding to the mutated G339 and G440 are highlighted by a dashed box. (B) Segmentally labelled BRD4(1, 2) with U-[^2^H,^15^N] and ^13^CH_3_ ILV methyl labelled BRD4^C^ (red).

NMR spectroscopy was used to validate the fold of the segmentally labelled sample. The 1D experiments showed similar profiles for wildtype and mutant forms of BRD4 in the amide region (data not shown). Moreover, the 2D ^1^H-^15^N TROSY HSQC of the segmentally labelled BRD4(1, 2) [V335L, S338T, Q339G, Q440G] showed good overlap with the uniformly [^2^H, ^15^N]-labelled wildtype BRD4(1, 2) (Fig 3A), thus confirming that the native and the tetra-mutant forms adopt the same global fold. The mutations are expected to have a negligible effect on BRD4(1) considering that the ligation mutations are located towards the C-terminus of the unstructured inter-domain linker and over 150 residues away from this bromodomain, which has a fold shown to be stable despite large structural rearrangements of tandem domain proteins [48, 49].

^1^H^N^ and ^15^N backbone assignments were obtained by transferring assignments from the isolated BRD4^C^[15] (see Materials & Methods). In the segmentally labelled protein, resonance assignments for 97% of the main-chain atoms were transferred from the isolated BRD4(2) (H388, L446 and D448 remained unassigned)(Fig 3B). For most of the residues, the weighted average ^1^H^N^ and ^15^N chemical shift differences between the uniformly and segmentally labelled samples are considerably smaller than 0.1 ppm, with the exception of four residues (I394, N428, Q447 and R453) that showed chemical shift changes larger than 0.1 ppm (*δ*_*ave*_ = 0.115–0.134 ppm)(S5 Fig). In addition, V449 was absent in our tandem domain spectrum but did appear in the fusion product. Given the small number of chemical shifts affected we concluded that the mutations that were introduced in order to make SrtA-mediated ligation possible occur in an unstructured region of the inter-domain linker and have negligible impact on the overall fold of BRD4(2). Of the eight residues mentioned above, five (L446, Q447, D448, V449 and R453) are clustered on the C-terminal α-helix. This suggests, that these are in close proximity to the linker in the three-dimensional structure of BRD4(2).

This spectrum serves as a good illustration of the positive effects of segmental labelling as the removal of strong signal from the unstructured linker in the crowded region of the spectrum greatly helps interpretation (Fig 3B).

**Fig 4:**
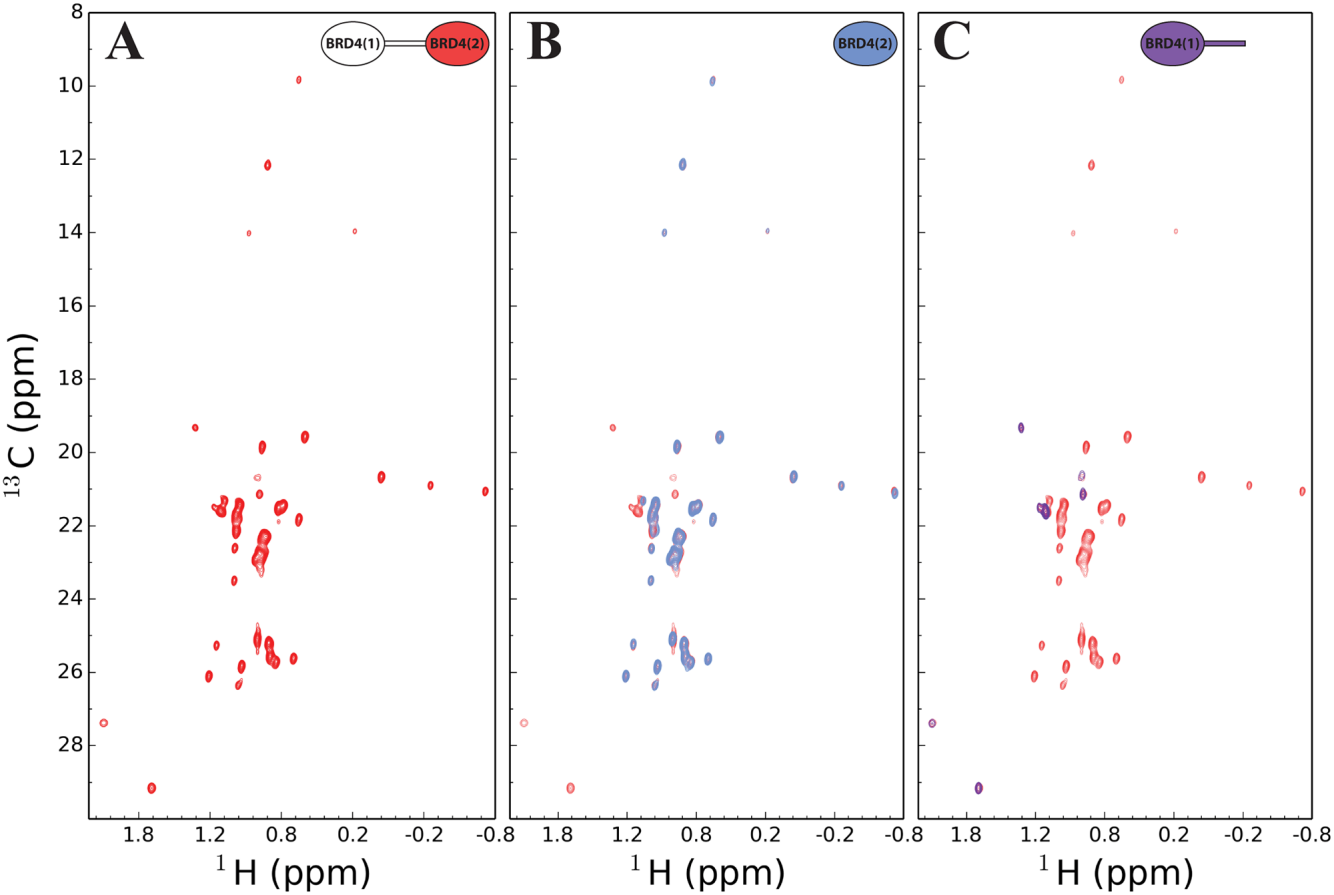
Methyl-TROSY of segmentally labelled BRD4(1, 2). (A) ^1^H-^13^C SOFAST TROSY HMQC of segmentally labelled BRD4(1, 2) with U-[^2^H,^15^N] and ^13^CH_3_ ILV methyl labelled BRD4^C^ (red). (B) Spectral overlay of (A) with isolated U-[^2^H,^15^N] and ^13^CH_3_ ILV methyl labelled BRD4^C^ (blue). (C) Spectral overlay of (A) with natural abundance BRD4^NL^ (purple).

The ^1^H-^13^C methyl-TROSY HMQC spectra acquired with the SOFAST [28, 29] implementation on the segmentally isotope labelled BRD4(1, 2) containing U-[^2^H,^15^N] isotopes and ILV methyl labelling on BRD4^C^ were of excellent quality with resonances appearing at the expected chemical shift range for Ile, Leu and Val methyls (Fig 4A). The C-terminal bromodomain consists of 4 Ile, 7 Leu and 7 Val residues. The tandem BRD4(1, 2) construct, however, consists of 18 Ile, 24 Leu and 27 Val residues. For an uniformly ILV labelled BRD4(1, 2) sample, the ^1^H-^13^C correlation spectra would contain a three to five-fold increase of methyl probes in the spectrum and would thus be less trivial to interpret. It can be seen that the segmentally labelled sample already suffers from minor overlap in the centre of the spectrum (Fig 4A), and this would worsen in a uniformly labelled ILV sample creating ambiguity.

In addition to the expected peaks, the ^1^H-^13^C correlation spectrum showed a complex set of additional peaks (Fig 4). A sample containing unlabelled BRD4^NL^ confirmed that these peaks arise from natural abundance ^13^C (Fig 4C). These peaks are most likely arising from regions in the interdomain linker with inherently increased dynamics as these are expected to relax slower and thus appear despite the low natural abundance of ^13^C.

In summary, the good overlap between our ligated product and signal from the isolated BRD4(2) domain again suggest that the product is correctly folded.

### An application of segmental labelling: BRD4 inhibition through I-BET762

The excellent quality of the ^1^H-^13^C methyl-TROSY HMQC spectra arising from the segmentally isotope labelling scheme of the tandem domain BRD4 construct suggest that 2D NMR is feasible for ligand binding studies providing interpretation at residue resolution. In order to demonstrate this as a proof of concept, we explored the binding of the small-molecule inhibitor I-BET762 [1, 3, 50], which is currently in clinical trials as a treatment for NUT midline carcinoma, to our segmentally labelled tandem domain BRD4.

**Fig 5:**
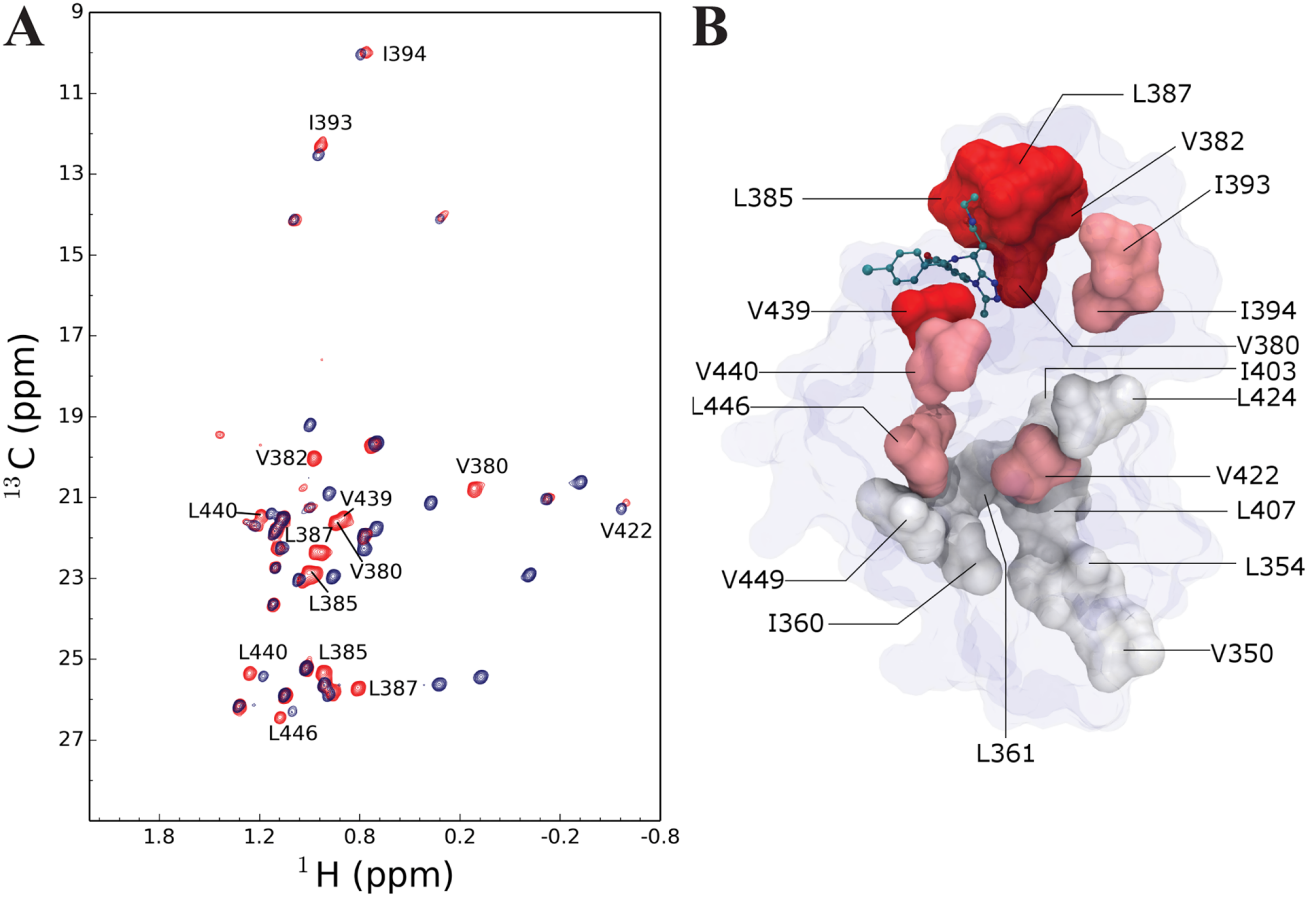
Titration of I-BET762 to segmentally labelled BRD4. (A) ^1^H-^13^C SOFAST TROSY HMQC of 12 μM segmentally labelled BRD4(1, 2) with U-[^2^H,^15^N] and ^13^CH_3_ ILV methyl labelled BRD4^C^ in apo (red) and in complex with 36 μM I-BET762 (blue). Residues undergoing significant changes in chemical shifts upon binding of I-BET762 are labelled. (B) Structure of BRD4(2) (blue envelope, PDB:2YEM) with surface representation of ILV residues colour coded according to whether they have undergone a large (red) small (pink) or no change (white) in chemical shift upon binding of I-BET762. I-BET762 is shown in a bond representation in its expected binding site based on a structural alignment with the structure of the complex BRD2(1):I-BET762 (PDB:2YEK).

Fig 5A shows ^1^H-^13^C SOFAST methyl-TROSY HMQC spectra of segmentally labelled tandem domain BRD4 in the apo and ligand bound states.

The spectra shown here were obtained using a 12 μM protein sample and were acquired in 10.5 h, although sufficient quality spectra for ligand binding studies, albeit with lower resolution and sensitivity, can be achieved in about 1.5 h (data not shown). The low protein concentration and comparatively low time requirement for useful binding studies is promising as it would allow consumption of less resources in the context of screening compounds. In addition, the low protein concentrations allow achievement of high saturation of the target whilst maintaining low ligand concentrations which will be desirable for the screening compounds of low solubility. Segmental labelling is essential to this method as without it, the additional ILV peaks from the BRD4(1) and linker domains would cause large amounts of overlap and make the methyl spectrum difficult to interpret.

^1^H-^13^C methyl-TROSY HMQC spectra at 12 μM protein concentration in the absence of I-BET762 and at a 36 μM compound concentration were acquired (Fig 5A). This ratio was used in order to ensure complete binding of both bromodomains; the reported affinities of I-BET762 to BRD4(1) and BRD4(2) are 95 nM and 65 nM, respectively [51]. Fortunately, the assignments of the apo BRD4(2) have been published previously [15] (BMRB ID: 15057) and were transferred to our methyl-spectrum. The potent inhibitor I-BET762 binds BRD4 in the slow-exchange regime, on the NMR timescale, making it laborious to assign peaks in the bound spectrum. However, peaks that disappear as the compound is added can be easily identified and therefore provide useful probes of interactions (Fig 5A). The disappearing peaks can be split into three categories. Residues V350, L354, I360, L361, I403, L407, L424 and V449 showed no changes in chemical shifts upon titration (category I).

Residues I393, I394, V422, L440 and L446 had peaks in the apo spectrum that disappeared in the presence of I-BET762; however nearby shifted peaks were observed in the bound form highly suggesting that these peaks correspond to the same residue with minor chemical shift changes upon titration (category II).

Residues V380, V382, L385, L387 and V439 experience a larger change in chemical shift upon titration (category III).

Fig 5B shows the location of the ILV residues on BRD4(2). As expected, residues in category III are located in close proximity to the binding pocket of BRD4(2), whereas residues in category II are located further away, but probe the presence of the compound as observed with minor chemical shift changes, while residues in category I are not affected by I-BET762 and are located far from the binding pocket. In summary, we have shown that the binding of I-BET762 to BRD4(2) can be probed at great detail using a segmentally labelled tandem domain BRD4 construct. Furthermore, using the SOFAST implementation good quality spectra can be acquired within a short period of time and using low quantities of labelled protein. This methodology can be used to confirm the binding of compounds to the individual domains of BRD4.

## Conclusion

At present, biophysical studies on BRD4(1, 2) are carried out using isolated BRD4(1) or BRD4(2) [15, 48, 52, 53]. Although this approach simplifies interpretation it may miss crucial bromodomain-bromodomain interactions, bromodomain-linker interactions and any interactions ligands may form with multiple domains. In order to address this, alternative strategies are required such as the use of BRD4(1, 2) mutants, where one acetyl-lysine binding pocket in the tandem domain BRD4(1, 2) is compromised [54] or the engineering of specificity for an inhibitor of a single bromodomain [51]. In this study the sequence of our ligated BRD4 differs from that of the wild type by four amino acids, however, the minor changes observed in the NMR spectra indicate that this does not significantly affect the protein, which is consistent with the mutations being located in a linker region predicted to be flexible based on strong resonance intensities and predominantly random coil chemical shifts in the ^1^H-^15^N TROSY HSQC spectra.

We therefore believe this system is an excellent model for BRD4 in its natural state and provides a powerful method to gather information about how the individually labelled domains interact in the context of the tandem domain protein and thus enabling a new avenue in the development of inhibitors with alternative binding modes. In addition, this system could possibly be further improved by the use of an engineered SrtA mutant with modified substrate specificity, reducing the number of mutations introduced to the final product [55]. Although we only demonstrated the segmental labelling of BRD4(2), it is trivial to design a construct that would allow for segmental labelling of BRD4(1), where the SrtA recognition sequence would be placed at the N-terminal region of the linker instead the C-terminal. Furthermore, the methods employed to design and produce segmentally labelled constructs using SrtA are applicable to other BRD4 constructs. For instance, the role of phosphorylation at the NPS site and its effects on the bromodomains could be probed with segmental labelling and 2D NMR spectroscopy. Using a segmental labelling approach will allow for studies without deuteration of all domains and our segmental methyl labelling scheme allows us to probe ligand binding rapidly, at low protein concentration and with sufficient resolution to determine whether the binding is specific. This type of labelling scheme will be key to understanding the tandem domain as a whole and provide a versatile tool in the development of inhibitors against this important biological target.

## Supporting Information

S1 Fig Illustration of simultaneous TEV cleavage of BRD4^C^ and SrtA-mediated ligation. In reaction condition A, 19 μM BRD4^NL^ were reacted with 78 μM uncleaved BRD4^C^ in the presence of 76 μM SrtA and 2.6 μM TEV protease in 50 mM Tris (pH 7.5), 150 mM NaCl and 1 mM TCEP. Condition B was identical except that SrtA concentration was 7.6 μM. Reactions were carried out at room temperature and timepoints were taken after 0 h, 2 h and 21 h reaction time. Reactions were stopped by addition of SDS running buffer and denaturation at 90°C.
(DOCX)

S2 Fig Illustration of differences in yield between reactions carried out in open or closed systems. Reactions were carried out between 18 μM BRD4^NL^ and 36 μM BRD4^C^ in the presence of 18 μM SrtA. Open system reaction was carried out in a 10 kDa cut-off concentrator at 21°C with centrifugation at 2000g, reaction volume was topped up every 10 min. Closed system reaction was carried out in an Eppendorf, without centrifugation, at 21°C. Buffer was 150 mM NaCl, 50 mM Tris (pH 7.5) and 1 mM TCEP. Samples were taken at 0, 0.5, 1, 2, 3, 4, 5 and 6 h reaction time. Signal is given as band intensity as a percentage of the total signal present in each lane.
(DOCX)

S3 Fig Illustration of differences in yield between reactions carried out in D_2_O and H_2_O. Reactions were carried out between 18 μM BRD4^NL^ and 36 μM BRD4^C^ in the presence of 18 μM SrtA. Reactions were carried out at room temperature. Buffers were 150 mM NaCl, 50 mM Tris (pH 8.0 or pD 8.0) and 1 mM TCEP in H_2_O or D_2_O. Samples were taken at 0, 0.5, 1, 2, 3, 4, 5 and 21 h reaction time. Signal is given as band intensity as a percentage of the total signal present in each lane.
(DOCX)

S4 Fig Comparison of yields obtained in an open system in D_2_O compared to H_2_O. Reactions were carried out between 18 μM BRD4^NL^ and 36 μM BRD4^C^ in the presence of 18 μM SrtA. Reactions were carried out in a 10 kDa cut-off concentrator at 21°C with centrifugation at 2000g, reaction volume was topped up every 10 min. Buffers were 150 mM NaCl, 50 mM Tris (pH 7.5 or pD 7.5) and 1 mM TCEP in H_2_O or D_2_O. Samples were taken at 0, 0.5, 1, 2, 3, 4, 5 and 6 h reaction time. Signal is given as band intensity as a percentage of the total signal present in each lane.
(DOCX)

S5 FigWeighted average of the ^1^H^N^ and ^15^N chemical shift differences for residues in the C-terminal bromodomain between wildtype and mutant BRD4.(A) Average chemical shift differences reported for BRD4(2) between wildtype tandem domain BRD4(1, 2) and BRD4(1, 2)[V335L, S338T, Q339G, Q440G] as a function of residue number. (B) Average chemical shift differences reported for BRD4(2) between wildtype isolated BRD4(2) and BRD4(1, 2)[V335L, S338T, Q339G, Q440G] as a function of residue number. Average chemical shift changes were obtained using the equation $\delta_{ave}=\sqrt{{\left(\Delta \delta H\right)}^{2}+{\left(\frac{1}{5}\Delta \delta N\right)}^{2}}$.(DOCX)

S1 Table Maximum signal from a set of experiments carried out simultaneously at different pH. Reactions were carried out between 18 μM BRD4^NL^ and 36 μM BRD4^C^ in the presence of 18 μM SrtA. Reactions were carried out at room temperature. Buffers were 150 mM NaCl, 50 mM Tris (pH 7.5, 8.0, 8.5 or pD 8.0) or 50 mM phosphate (pH 6.5 and 7.0) and 1 mM TCEP in H_2_O or D_2_O. Samples were taken at 0, 0.5, 1, 2, 3, 4, 5 and 21 h reaction time. Signal is given as band intensity as a percentage of the total signal present in each lane.
(DOCX)
