# Supplementary material for: Segmental Isotope Labelling of an Individual Bromodomain of a Tandem Domain BRD4 Using Sortase A
Source: PLoS One. 2016 Apr 29;11(4):e0154607. doi: 10.1371/journal.pone.0154607 (PMC4851411; doi:10.1371/journal.pone.0154607)
Supplement: S1 Fig — In reaction condition A, 19 μM BRD4NL were reacted with 78 μM uncleaved BRD4C in the presence of 76 μM SrtA and 2.6 μM TEV protease in 50 mM Tris (pH 7.5), 150 mM NaCl and 1 mM TCEP. Condition B was identical except that SrtA concentration was 7.6 μM. Reactions were carried out at room temperature and timepoints were taken after 0 h, 2 h and 21 h reaction time. Reactions were stopped by addition of SDS running buffer and denaturation at 90°C. (DOCX) [file pone.0154607.s001.docx]

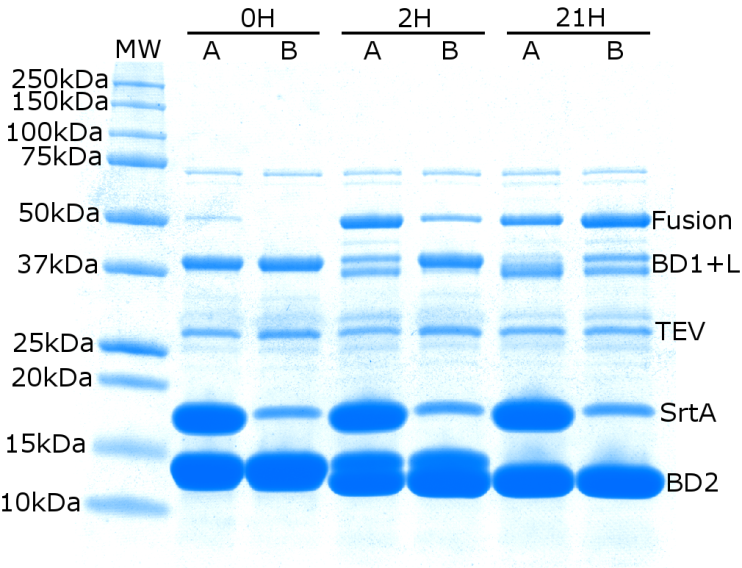


Figure S1: Illustration of simultaneous TEV cleavage of BRD4^C^ and SrtA-mediated ligation. In reaction condition A, 19 µM BRD4^NL^ were reacted with 78 µM uncleaved BRD4^C^ in the presence of 76 µM SrtA and 2.6 µM TEV protease in 50 mM Tris (pH 7.5), 150 mM NaCl and 1 mM TCEP. Condition B was identical except that SrtA concentration was 7.6 µM. Reactions were carried out at room temperature and timepoints were taken after 0 h, 2 h and 21 h reaction time. Reactions were stopped by addition of SDS running buffer and denaturation at 90°C.
