# Supplementary material for: Segmental Isotope Labelling of an Individual Bromodomain of a Tandem Domain BRD4 Using Sortase A
Source: PLoS One. 2016 Apr 29;11(4):e0154607. doi: 10.1371/journal.pone.0154607 (PMC4851411; doi:10.1371/journal.pone.0154607)
Supplement: S2 Fig — Reactions were carried out between 18 μM BRD4NL and 36 μM BRD4C in the presence of 18 μM SrtA. Open system reaction was carried out in a 10 kDa cut-off concentrator at 21°C with centrifugation at 2000g, reaction volume was topped up every 10 min. Closed system reaction was carried out in an Eppendorf, without centrifugation, at 21°C. Buffer was 150 mM NaCl, 50 mM Tris (pH 7.5) and 1 mM TCEP. Samples were taken at 0, 0.5, 1, 2, 3, 4, 5 and 6 h reaction time. Signal is given as band intensity as a percentage of the total signal present in each lane. (DOCX) [file pone.0154607.s002.docx]

Figure S2: Illustration of differences in yield between reactions carried out in open or closed systems. Reactions were carried out between 18 µM BRD4^NL^ and 36 µM BRD4^C^ in the presence of 18 µM SrtA. Open system reaction was carried out in a 10 kDa cut-off concentrator at 21°C with centrifugation at 2000g, reaction volume was topped up every 10 min. Closed system reaction was carried out in an Eppendorf, without centrifugation, at 21°C. Buffer was 150 mM NaCl, 50 mM Tris (pH 7.5) and 1 mM TCEP. Samples were taken at 0, 0.5, 1, 2, 3, 4, 5 and 6 h reaction time. Signal is given as band intensity as a percentage of the total signal present in each lane.
