# Supplementary material for: Segmental Isotope Labelling of an Individual Bromodomain of a Tandem Domain BRD4 Using Sortase A
Source: PLoS One. 2016 Apr 29;11(4):e0154607. doi: 10.1371/journal.pone.0154607 (PMC4851411; doi:10.1371/journal.pone.0154607)
Supplement: S3 Fig — Reactions were carried out between 18 μM BRD4NL and 36 μM BRD4C in the presence of 18 μM SrtA. Reactions were carried out at room temperature. Buffers were 150 mM NaCl, 50 mM Tris (pH 8.0 or pD 8.0) and 1 mM TCEP in H2O or D2O. Samples were taken at 0, 0.5, 1, 2, 3, 4, 5 and 21 h reaction time. Signal is given as band intensity as a percentage of the total signal present in each lane. (DOCX) [file pone.0154607.s003.docx]

Figure S3: Illustration of differences in yield between reactions carried out in D_2_O and H_2_O. Reactions were carried out between 18 µM BRD4^NL^ and 36 µM BRD4^C^ in the presence of 18 µM SrtA. Reactions were carried out at room temperature. Buffers were 150 mM NaCl, 50 mM Tris (pH 8.0 or pD 8.0) and 1 mM TCEP in H_2_O or D_2_O. Samples were taken at 0, 0.5, 1, 2, 3, 4, 5 and 21 h reaction time. Signal is given as band intensity as a percentage of the total signal present in each lane.
