# Supplementary material for: Segmental Isotope Labelling of an Individual Bromodomain of a Tandem Domain BRD4 Using Sortase A
Source: PLoS One. 2016 Apr 29;11(4):e0154607. doi: 10.1371/journal.pone.0154607 (PMC4851411; doi:10.1371/journal.pone.0154607)
Supplement: S4 Fig — Reactions were carried out between 18 μM BRD4NL and 36 μM BRD4C in the presence of 18 μM SrtA. Reactions were carried out in a 10 kDa cut-off concentrator at 21°C with centrifugation at 2000g, reaction volume was topped up every 10 min. Buffers were 150 mM NaCl, 50 mM Tris (pH 7.5 or pD 7.5) and 1 mM TCEP in H2O or D2O. Samples were taken at 0, 0.5, 1, 2, 3, 4, 5 and 6 h reaction time. Signal is given as band intensity as a percentage of the total signal present in each lane. (DOCX) [file pone.0154607.s004.docx]

Figure S4: Comparison of yields obtained in an open system in D_2_O compared to H_2_O. Reactions were carried out between 18 µM BRD4^NL^ and 36 µM BRD4^C^ in the presence of 18 µM SrtA. Reactions were carried out in a 10 kDa cut-off concentrator at 21°C with centrifugation at 2000g, reaction volume was topped up every 10 minutes. Buffers were 150 mM NaCl, 50 mM Tris (pH 7.5 or pD 7.5) and 1 mM TCEP in H_2_O or D_2_O. Samples were taken at 0, 0.5, 1, 2, 3, 4, 5 and 6 h reaction time. Signal is given as band intensity as a percentage of the total signal present in each lane.
