# Supplementary material for: Segmental Isotope Labelling of an Individual Bromodomain of a Tandem Domain BRD4 Using Sortase A
Source: PLoS One. 2016 Apr 29;11(4):e0154607. doi: 10.1371/journal.pone.0154607 (PMC4851411; doi:10.1371/journal.pone.0154607)
Supplement: S5 Fig — (A) Average chemical shift differences reported for BRD4(2) between wildtype tandem domain BRD4(1, 2) and BRD4(1, 2)[V335L, S338T, Q339G, Q440G] as a function of residue number. (B) Average chemical shift differences reported for BRD4(2) between wildtype isolated BRD4(2) and BRD4(1, 2)[V335L, S338T, Q339G, Q440G] as a function of residue number. Average chemical shift changes were obtained using the equation δave=(ΔδH)2+(15ΔδN)2. (DOCX) [file pone.0154607.s005.docx]

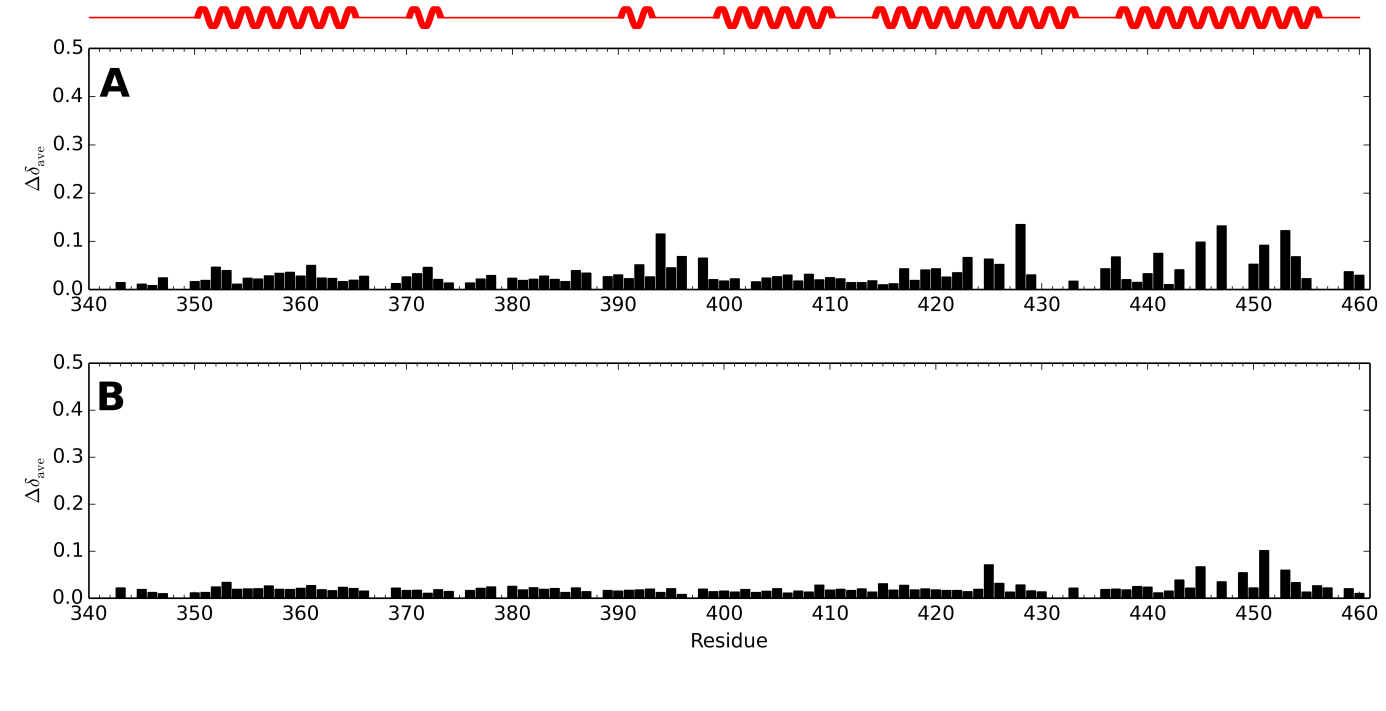


Figure S5: Weighted average of the ^1^H^N^ and ^15^N chemical shift differences for residues in the C-terminal bromodomain between wildtype and mutant BRD4. (A) Average chemical shift differences reported for BRD4(2) between wildtype tandem domain BRD4(1,2) and BRD4(1,2)[V335L, S338T, Q339G, Q440G] as a function of residue number. (B) Average chemical shift differences reported for BRD4(2) between wildtype isolated BRD4(2) and BRD4(1,2)[V335L, S338T, Q339G, Q440G] as a function of residue number. Average chemical shift changes were obtained using the equation $\sqrt{\boldsymbol{(\Delta\delta H)}^{\boldsymbol{2}}\boldsymbol{+}{\boldsymbol{(}\frac{\boldsymbol{1}}{\boldsymbol{5}}\boldsymbol{\Delta\delta N)}}^{\boldsymbol{2}}}$.
