# Supplementary material for: Segmental Isotope Labelling of an Individual Bromodomain of a Tandem Domain BRD4 Using Sortase A
Source: PLoS One. 2016 Apr 29;11(4):e0154607. doi: 10.1371/journal.pone.0154607 (PMC4851411; doi:10.1371/journal.pone.0154607)
Supplement: S1 Table — Reactions were carried out between 18 μM BRD4NL and 36 μM BRD4C in the presence of 18 μM SrtA. Reactions were carried out at room temperature. Buffers were 150 mM NaCl, 50 mM Tris (pH 7.5, 8.0, 8.5 or pD 8.0) or 50 mM phosphate (pH 6.5 and 7.0) and 1 mM TCEP in H2O or D2O. Samples were taken at 0, 0.5, 1, 2, 3, 4, 5 and 21 h reaction time. Signal is given as band intensity as a percentage of the total signal present in each lane. (DOCX) [file pone.0154607.s006.docx]

| Maximum signal  (as percentage of total signal in lane) | |
| --- | --- |
| D_2_O pD 8.0 | 10.4% |
| H_2_O pH 6.5 | 5.4% |
| H_2_O pH 7.0 | 6.2% |
| H_2_O pH 7.5 | 7.1% |
| H_2_O pH 8.0 | 6.4% |
| H_2_O pH 8.5 | 4.5% |

Table S1: Maximum signal from a set of experiments carried out simultaneously at different pH. Reactions were carried out between 18 µM BRD4^NL^ and 36 µM BRD4^C^ in the presence of 18 µM SrtA. Reactions were carried out at room temperature. Buffers were 150 mM NaCl, 50 mM Tris (pH 7.5, 8.0, 8.5 or pD 8.0) or 50mM phosphate (pH 6.5 and 7.0) and 1 mM TCEP in H_2_O or D_2_O. Samples were taken at 0, 0.5, 1, 2, 3, 4, 5 and 21 h reaction time. Signal is given as band intensity as a percentage of the total signal present in each lane.
